# HERV-K and HERV-H Env Proteins Induce a Humoral Response in Prostate Cancer Patients

**DOI:** 10.3390/pathogens11010095

**Published:** 2022-01-14

**Authors:** Maria Antonietta Manca, Tatiana Solinas, Elena Rita Simula, Marta Noli, Stefano Ruberto, Massimo Madonia, Leonardo A. Sechi

**Affiliations:** 1 Dipartimento di Scienze Biomediche, Università di Sassari, 07100 Sassari, Italy; m.anto.manca@gmail.com (M.A.M.); simulaelena@gmail.com (E.R.S.); martanoli@outlook.it (M.N.); ruberto.ste@gmail.com (S.R.); 2 Dipartimento di Scienze Mediche, Chirurgiche e Sperimentali, Università di Sassari, 07100 Sassari, Italy; tatiana.solinas@tiscali.it (T.S.); madonia@uniss.it (M.M.); 3 Struttura Complessa di Urologia, Azienda Ospedaliera Universitaria, 07100 Sassari, Italy; 4 Struttura Complessa di Microbiologia e Virologia, Azienda Ospedaliera Universitaria, 07100 Sassari, Italy

**Keywords:** prostate cancer, HERV-H, HERV-K, humoral response, ELISA

## Abstract

A higher expression of human endogenous retroviruses (HERVs) has been associated with several malignancies, including prostate cancer, implying a possible use as a diagnostic or prognostic cancer biomarker. For this reason, we examined the humoral response against different epitopes obtained from the envelope protein of HERV-K (HERV-K env-su_19–37_, HERV-K env-su_109–126_), HERV-H (HERV-H env-su_229–241_, HERV-H env_387–399_) and HERV-W (HERV-W env-su_93–108_, HERV-W env-su_248–262_) in the plasma of patients affected by prostate cancer (PCa), and compared to that of benign prostate hyperplasia (BPH) and a borderline group of patients with atypical small acinar proliferation (ASAP) and prostate intraepithelial neoplasia (PIN) and healthy controls. A significant antibody response was observed against HERV-K env-su_109–126_ (*p* = 0.004) and HERV-H env-su_229–241_ (*p* < 0.0001) in PCa patients compared to HCs, BPH and borderline cohorts, whilst no significance difference was found in the antibodies against HERV-W env-su_93–108_ and HERV-W env-su_248–262_ in patients with PCa. Our results provided further proof of the association between HERV-K and PCa and added new evidence about the possible involvement of HERV-H in PCa pathogenesis, highlighting their possibility of being used as biomarkers of the disease.

## 1. Introduction

Prostate cancer (PCa) is the most common cause of death by cancer in the male population. The incidence rates appear to change depending on race, ethnicity, and geography, but also aging, family history, obesity, and diet [1,2,3]. Several studies pointed out both chronic inflammation and genitourinary infections as possible contributors in prostatic carcinogenesis and tumor progression [4,5]. PCa is a heterogenous disease, and one of the challenges is the inability of the current diagnostic tests, including the protease-specific antigen (PSA) screening and histopathological grading system, to distinguish between indolent and aggressive tumors [6]. The human genome accounts for approximately 8% of sequences derived from endogenous retroviruses (ERVs). To date, these sequences are remnants of exogenous retroviral infections of the germ cell line which guaranteed their vertical transmission in a Mendelian fashion [7]. HERVs are not infectious, and their pathogenicity has been dampened due to the accumulation of deleterious mutations, deletions, and epigenetic modifications, such as DNA methylation, which contributed to the repression of HERV expression [8]. However, evidence confirms that, in specific circumstances, HERVs sequences undergo a process of reactivation. The role of some HERV envelope (env) proteins is well known, both in health tissue and disease [9,10,11]. Different mechanisms have been proposed about the role of HERVs in cancer and in the transformation of benign cells. First, HERVs possess 2 long terminal repeats (LTRs), since they share a typical retroviral genomic structure (5′LTR-gag, pro, pol, env-3′LTR), which can alter the expression of host cell genes, leading to uncontrolled cell proliferation. Second, some HERVs, such as HERV-K, encode proteins with oncogenic properties. HERV-K(HML2) has been recognized as the most recently acquired and active member of the HERVs family, therefore, it retained complete open reading frames (ORFs) and the capability to encode functional proteins [12,13]. HERV-K(HML-2) env gene is characterized by the presence or absence of a 292 bp deletion, which leads to the expression of two splicing variants, Np9 and Rec, respectively [14,15]. Np9 plays an intriguing role in the co-activation of β-catenin, ERK, Akt and Notch1 promoting the growth of human leukemia stem/progenitor cells [16]. Both Np9 and Rec have been shown to interact, physically and functionally, with the promyelocytic zinc finger (PLZF) tumor suppressor, and inhibit its role as a transcriptional repressor, leading to c-Myc overexpression and alterations in the expression patterns of c-Myc target genes [17]. Despite HERV-K env gene expression in healthy tissues [18,19], its reactivation has been documented in several malignancies, such as melanoma, leukemia, breast cancer, lymphoma, and teratocarcinoma [20,21,22,23,24]. Recently, Sgarbi Reis et al. [25] reported the increased expression of HERV-K gag in PCa tissues, and increased levels of HERV-K gag autoantibodies in a subset of patients with advanced PCa compared to healthy controls (HCs). An increase in HERV-K gag mRNA expression was also found in the peripheral blood mononuclear cells (PBMC) of older men and smokers with PCa [26]. With regard to the epigenetic changes during tumorigenesis, several studies reported an alteration in the expression of both HERV-W and HERV-H, and a specific immune response directed against the two retroviruses [27,28,29]. The aim of this study was to evaluate the presence of autoantibodies against different epitopes derived from the envelope protein of HERV-K, HERV-W and HERV-H in the plasma of patients with PCa compared to healthy controls (HCs), a cohort of patients with benign prostate hyperplasia (BPH), and a borderline group of patients with atypical small acinar proliferation (ASAP) and prostate intraepithelial neoplasia (PIN). 

## 2. Results

**Figure 1:**
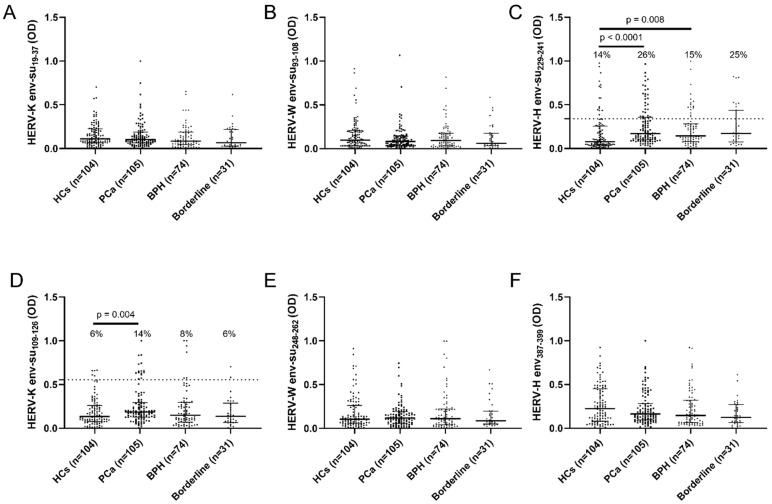
Indirect ELISA assay to detect antibodies against HERV env-derived peptides. Plasma samples from HCs, PCa, BPH and borderline groups were tested to detect circulating antibodies against HERV-K env-su_19–37_ (**A**), HERV-K env-su_109–126_ (**D**), HERV-W env-su_93–108_ (**B**), HERV-W env-su_248–262_ (**E**), HERV-H env-su_229–241_ (**C**) and HERV-H env_387–399_ (**F**). The black bars represent median plus interquartile range, whether the dotted lines represent the cut-off values for seropositivity obtained after ROC analysis.

The detection of antibodies against the selected highly immunogenic peptides derived from different portions of the envelope protein of HERV-K, HERV-W and HERV-H was carried out by an indirect ELISA assay within the HCs, PCa, BPH and the borderline cohorts. Fifteen out of 105 (14%) patients with PCa (*p* = 0.004), 6 out of 74 (8%) patients with PBH, 2 out of 31 (6%) patients of the borderline group, and 6 out of 104 (6%) of the healthy controls were seropositive against HERV-K env-su_109–126_ (Figure 1D). Twenty-eight out of 105 (26%) patients with PCa (*p* < 0.0001), 16 out of 74 (15%) patients with PBH (*p* = 0.008)—8 out of 31 (25%) patients of the borderline group and 15 out of 104 (14%) of healthy controls—were seropositive against HERV-H env-su_229–241_ (Figure 1C). No statistically significant difference in the humoral response against HERV-K env-su_19–37_ (Figure 1A), HERV-W env-su_93–108_ (Figure 1B), HERV-W env-su_248–262_ (Figure 1E) and HERV-H env_387–399_ (Figure 1F) has been found between PCa patients and HCs, as well as between PCa and the other disease groups, BPH and borderline. 

**Figure 2:**
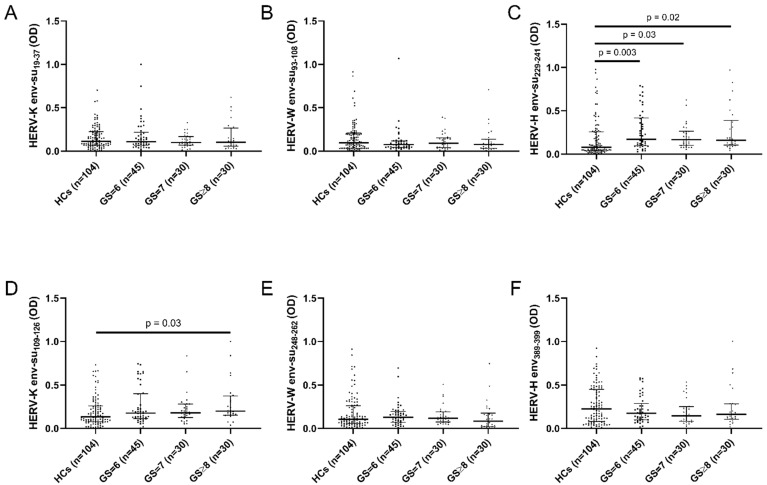
Stratification of PCa patients based on Gleason grading system and analysis of antibody response against HERV-K env-su_19–37_ (**A**), HERV-K env-su_109–126_ (**D**), HERV-W env-su_93–108_ (**B**), HERV-W env-su_248–262_ (**E**), HERV-H env-su_229–241_ (**C**) and HERV-H env_387–399_ (**F**). The black bars represent median plus interquartile range.

To determine whether the humoral response against HERV env-derived peptides was influenced by the patient grading system and the cancer progression, we stratified the population of PCa patients according to the Gleason grading system. The analysis exhibited a statistically significant difference in the antibody response against HERV-K env-su_109–126_ (Figure 2D) between HCs and the GS ≥ 8 group (*p* = 0.03). A significant difference was also found in the antibody response directed against HERV-H env-su_229–241_ (Figure 2F) between HCs and patients with GS = 6 (*p* = 0.001), GS = 7 (*p* = 0.03) and GS ≥ 8 (*p* = 0.01). However, no differences were found in the antibody levels among the various GS classes against HERV-H env-su_229–241_, indicating that the immune response directed against the envelope protein of HERV-H was not affected by the Gleason grading and the level of differentiation of prostate tissue. No differences were found in the humoral response against HERV-K env-su_19–37_ (Figure 2A), HERV-W env-su_93–108_ (Figure 2B), HERV-W env-su_248–262_ (Figure 2E) and HERV-H env_387–399_ (Figure 2F). 

**Figure 3:**
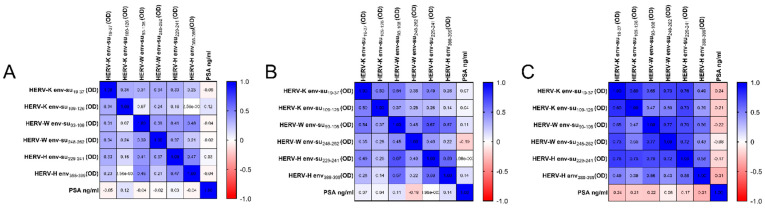
The heatmap shows the Spearman’s correlation coefficients among HERV env-derived peptides and PSA plasmatic levels in PCa (**A**), BPH (**B**) and borderline (**C**) cohorts.

In Figure 3, Spearman’s correlation shows that PSA plasmatic levels are not correlated to the humoral response against HERV env-derived peptides; not only in patients with PCa (Figure 3A), but also in patients with PBH (Figure 3B), and in patients of the borderline group (Figure 3C). In PCa patients (Figure 3A), the results show the following correlations between HERV-K env-su_19–37_ and HERV-K env-su_109–126_ (r = 0.34, *p* = 0.0004), HERV-W env-su_93–108_ (r = 0.31, *p* = 0.001), HERV-W env-su_248–262_ (r = 0.34, *p* = 0.0004), HERV-H env-su_229–241_ (r = 0.33, *p* = 0.0005) and HERV-H env_387–399_ (r = 0.23, *p* = 0.01); low correlations were found between HERV-K env-su_109–126_ and HERV-W env-su_93–108_ (r = 0.07, *p* = ns), HERV-W env-su_248-262_ (r = 0.24, *p* = 0.01), HERV-H env-su_229-241_ (r = 0.16, *p* = ns) and HERV-H env_387-399_ (r = −0.003, *p* = ns); low correlations were also found between HERV-W env-su_93-108_ and HERV-W env-su_248-262_ (r = 0.39, *p* < 0.0001), HERV-H env-su_229-241_ (r = 0.41, *p* < 0.0001), HERV-H env_387-399_ (r = 0.46, *p* < 0.0001) and between HERV-W env-su_248-262_ and HERV-H env-su_229-241_ (r = 0.37, *p* = 0.0001), HERV-H env_387-399_ (r = 0.21, *p* = 0.02). An r correlation coefficient of 0.47 was found between HERV-H env-su_229-241_ HERV-H env_387-399_ (*p* < 0.0001). In BPH patients (Figure 3B), we found a correlation between HERV-K env-su_19–37_ and HERV-K env-su_109–126_ (r = 0.50, *p* < 0.0001), HERV-W env-su_93–108_ (r = 0.54, *p* < 0.0001), HERV-W env-su_248–262_ (r = 0.35, *p* = 0.002), HERV-H env-su_229–241_ (r = 0.49, *p* < 0.0001) and HERV-H env_387–399_ (r = 0.28, *p* = 0.01). Low correlations were found between HERV-K env-su_109–126_ and HERV-W env-su_93–108_ (r = 0.37, *p* = 0.001), HERV-W env-su_248–262_ (r = 0.28, *p* = 0.01), HERV-H env-su_229–241_ (r = 0.26, *p* = 0.02); no significant correlation was found between HERV-K env-su_109–126_ and HERV-H env_387–399_ (r = 0.14, *p* = ns). A moderate correlation was found between HERV-W env-su_93–108_ and HERV-W env-su_248–262_ (r = 0.45, *p* < 0.0001), HERV-H env-su_229–241_ (r = 0.67, *p* < 0.0001), HERV-H env_387–399_ (r = 0.57, *p* < 0.0001). HERV-W env-su_248–262_ showed the following correlations with HERV-H env-su_229–241_ (r = 0.40, *p* < 0.0001), HERV-H env_387–399_ (r = 0.22, *p* = ns); whist, a moderate correlation was found between HERV-H env-su_229–241_ and HERV-H env_387–399_ (r = 0.59, *p* < 0.0001). In the Borderline group (Figure 3C), a high correlation was found between HERV-K env-su_19–37_ and HERV-K env-su_109–126_ (r = 0.80, *p* < 0.0001), HERV-W env-su_248–262_ (r = 0.73, *p* < 0.0001), HERV-H env-su_229–241_ (r = 0.76, *p* < 0.0001), and between HERV-W env-su_93–108_ and HERV-W env-su_248–262_ (r = 0.77, *p* < 0.0001), HERV-H env-su_229–241_ (r = 0.70, *p* < 0.0001). A high correlation was also found between HERV-H env-su_229–241_ and HERV-W env-su_248–262_ (r = 0.72, *p* < 0.0001). A moderate correlation was observed between HERV-K env-su_109–126_ and HERV-W env-su_93–108_ (r = 0.47, *p* = 0.007), HERV-W env-su_248–262_ (r = 0.50, *p* = 0.004), HERV-H env_387–399_ (r = 0.39, *p* = 0.02), between HERV-H env_387–399_ and HERV-W env-su_248–262_ (r = 0.43, *p* = 0.01), HERV-K env-su_19–37_ (r = 0.49, *p* = 0.005), HERV-W env-su_93–108_ (r = 0.56, *p* = 0.001), HERV-H env-su_229–241_ (r = 0.58, *p* = 0.007), and between HERV-K env-su_19–37_ and HERV-W env-su_93–108_ (r = 0.65, *p* = 0.0001).

## 3. Discussion

Over the years, several studies have investigated the possible involvement of HERV in different diseases, such as multiple sclerosis [30], amyotrophic lateral sclerosis [31], autoimmune diseases [9,32], and especially cancer [33,34,35,36]. The envelope protein of HERV family viruses is well known for its immunosuppressive properties and its role in the modulating transcription factors of cancer-associated pathways [16,37]. Regarding PCa, screening methods for diagnosis and prognosis are necessary for cancer management, and to date, healthcare has been using both invasive (biopsy) and low invasive (PSA screening) approaches in PCa diagnoses. Several studies used different approaches to investigate the role played by HERVs in cancer pathogenesis. Recently, Rezaei et al. reported elevated levels of HERV-K gag RNA and protein in malignant regions of the male prostate with PCa compared to matched benign regions [38]. Meanwhile, previous studies reported the presence of serum antibody against HERV-K gag in prostate cancer [25] and an increased expression of HERV-K env in the PBMCs of PCa patients, which appeared to be affected by age and smoking status [26]. Chie et al. instead reported the involvement of HERV-H in cancer immune evasion and its role in amplifying the epithelial-to-mesenchymal transition [39]. This latter study proved the ability of an HERV-H-derived peptide to increase the production of CCL19, and its role as a chemo attractive factor in recruiting an elevated number of immunosuppressive immune cells in HERV-H^+^ CCL19^+^ colon cancer tissues. In their work, Pérot et al. suggest a functional role for HERV-H in colorectal carcinogenesis, proving a correlation between HERV-H reactivation and clinical parameters, such as the presence of tumor cells in lymph nodes [40]. Here, we investigated the presence of a humoral response against highly immunogenic peptides derived from the envelope proteins of HERV-K, HERV-W, and HERV-H in the plasma of patients with PCa, BPH and a borderline cohort, given the easy accessibility of such body fluid and its advantage in using a specific HERV-directed immune response as a possible disease biomarker. We found that 14% of patients with PCa displayed higher antibody levels against HERV-K env-su_109–126_ compared to HCs, BPH and the borderline cohorts, while no differences were found in the humoral response against the peptides derived from the envelope protein of HERV-W (HERV-W en-su_93–108_ and HERV-W env-su_248–262_). We also found, for the first time, that 26% of patients with PCa displayed a strong antibody response against HERV-H env-su_229–241_ compared to HCs, BPH, and the borderline cohorts. In the BPH group, 14% of patients were found seropositive to HERV-H env-su_229–241_, though this result was not confirmed after a T-Fisher exact test. To deepen the significance of HERV-env-specific humoral response, we decided to stratify the population of PCa patients according to the Gleason score system, a powerful predictor of PCa prognosis. Our results pointed out the fact that the humoral response directed against HERV-H env-su_229–241_ did not appear to be correlated to the Gleason grading system and the tumor progression, since no significant differences were found among the GS-based groups, whilst the humoral response against HERV-K env-su_109–126_ appeared stronger in patients with a GS ≥ 8. These findings support the hypothesis of a potential role for HERV-K env and HERV-H env autoantibodies as prostate cancer biomarkers; it may be useful to help elucidate the diagnostic and prognostic value of HERV serum autoantibodies to further investigate the expression levels of the envelope protein of HERV-K and HERV-H in biopsy samples, as well as PBMC. 

## 4. Materials and Methods

### 4.1. Study Population and Blood Collection

This study was approved by the Ethical Committee of AOU Sassari, and all patients signed an informed consent form. The study population consisted of 105 patients with PCa, 74 patients with BPH, and a borderline group of 31 patients diagnosed with ASAP and PIN. The population of HCs consisted of 105 individuals matched by age. The PCa, BPH and borderline cohorts were recruited from the Urology Department of University Hospital, Sassari, while the HCs were recruited from the Transfusion Center, AOU, Sassari. Peripheral venous blood was collected at the time of the subject recruitment, and was collected in K^+^ -EDTA test tubes, both for patients and HCs. The plasma samples were isolated from whole blood by standard Ficoll–Histopaque (Sigma-Aldrich, St. Louis, MO, USA) gradient centrifugation and stored at −20 °C; thereafter, they were used to test the presence of autoantibodies against HERV env-derived peptides by an indirect ELISA assay. Clinical data about the patients and HCs are shown in Table 1. 

### 4.2. Highly Immunogenic Peptides

The peptides HERV-K env_19–37,_ HERV-K env_109–126_, HERV-W env_93–108_, HERV-W env_248–262_, HERV-H env_229–241_, HERV-H env_387–399_, were designed by using the Immune Epitope Database (IEBD) and synthesized at >95% purity (LifeTein, South Plainfield, NJ, USA). All peptides have been resuspended in DMSO and stored in single-use aliquots at −80 °C (Table 2).

### 4.3. Enzyme-Linked Immunosorbent Assay (ELISA)

Indirect ELISA has been performed to evaluate the presence of a specific humoral response against highly immunogenic peptides derived from the envelope protein of HERV-K, HERV-W, and HERV-H. Ninety-six-well Nunc immune-plates were incubated overnight at 4 °C with 0.05 M of carbonate–bicarbonate (pH 9.5, Sigma-Aldrich, St. Louis, MO, USA), and the respective peptides at 10 µg/mL. The plates were incubated for 1 h in a blocking solution with 5% non-fat dried milk (Sigma-Aldrich, St. Louis, MO, USA) and phosphate-buffered saline (PBS), then washed twice in a solution with PBS and 0.05% Tween-20 (PBS-T). Plasma samples were added at a 1:100 concentration and incubated for 2 h at room temperature. After a washing step, each plate was incubated for 1 h with 100 µL of PBS and an alkaline phosphate-conjugated goat anti-human IgG polyclonal antibody (1:1000, Sigma-Aldrich, St. Louis, MO, USA). After washing, each plate was washed in PBS-T and then incubated in milli-Q water and p-nitrophenyl phosphate (Sigma-Aldrich, St. Louis, MO, USA) for 10 min in a dark environment. The optical density was read at 405 nm using a SpectraMax Plus 384 microplate reader (Molecular Devices, Sunnyvale, CA, USA). Different negative controls were included: (1) an irrelevant peptide was present for each sample in all the experiments to verify non-specific binding; (2) one empty well (with no peptides); (3) one well where PBS was used instead of patient plasma. A competitive ELISA was performed by using HERV-K env-su_109–126_ and a different peptide (Annexin A2 _13–37_ LEGDHSTPPSAYGSVKAYTNFDAER, to which the patient was positive) in order to assess the specificity of the binding (Supplementary Figure S1). A statistically significant decrease of binding was observed only when the plasma of the positive patients was preincubated against the HERV-K-specific peptide. The results were normalized to a positive control included in all the experiments. The positive sample was previously tested for the reactivity to the selected peptides, and to an irrelevant peptide in order to verify the binding specificity. Moreover, competitive inhibition experiments were also performed, where the positive plasma before a normal ELISA was preadsorbed with the specific peptide or irrelevant peptides, in order to verify the reduction in absorbance [41,42]. 

### 4.4. Statistical Analysis

Data distribution was analyzed using the D’Agostino–Pearson omnibus normality test and the Shapiro–Wilk normality test. Non-parametric data were analyzed using the Mann–Whitney U test and the Kruskal–Wallis test with Dunn’s multiple comparisons test to compare the antibody levels against the different HERV env-derived peptides between two or more groups, respectively. The receiver-operating characteristic (ROC) was used to select a cut-off value to assess the sample positivity, which was confirmed through Fisher’s exact test. Sensitivity and specificity were chosen accordingly for all measured samples. Spearman’s correlation test was performed to evaluate the correlation between the humoral response against HERV env peptides and PSA levels. The level of statistical significance was set up as *p* < 0.05. Statistical analysis was carried out using GraphPad Prism 8.2.0 software (GraphPad Software, San Diego, CA, USA).

## Figures and Tables

**Table 1:** Demographic and clinical characteristics of patients and healthy controls.

	PCa (n = 105)	BPH (n = 74)	Borderline (n = 31)	HCs (n = 104)
Age (mean ± SD)	71.3 ± 8.2	66.3 ± 7.6	68.3 ± 7.4	60.1 ± 5.8
Serum PSA (ng/mL, mean ± SD)	33.6 ± 107.9	7.1 ± 6.9	6.0 ± 4.2	
≤4 ng/mL	16	24	8	
>4 ng/mL	86	48	21	
Unknown	1	1	1	
Gleason Score (GS)				
GS = 6	45			
GS = 7	30			
GS ≥ 8	26			
Unknown	4			

**Table 2:** Aminoacidic sequences of peptides used as antigens in the ELISA assay.

	Epitope Position	Epitope Sequence	UniProtKb Accession Number
HERV-K env-su_19–37_	19–37	VWVPGPTDDRCPAKPEEEG	O42043
HERV-K env-su_109–126_	109–126	RPKGKTCPKEIPKGSKNT	O42043
HERV-W env-su_93–108_	93–108	NPSCPGGLGVTVCWTY	Q9UQF0
HERV-W env-su_248–262_	248–262	NSQCIRWVTPPTQIV	Q9UQF0
HERV-H env-su_229–241_	229–241	LGRHLPCISLHPW	Q9N2J8
HERV-H env_387–399_	387–399	RVIPLIPLMVGLG	Q9N2J8

## Supplementary Materials

The following supporting information can be downloaded at: https://www.mdpi.com/article/10.3390/pathogens11010095/s1, Figure S1: Competitive ELISA assay.
