# Supplementary material for: HERV-K and HERV-H Env Proteins Induce a Humoral Response in Prostate Cancer Patients
Source: Pathogens. 2022 Jan 14;11(1):95. doi: 10.3390/pathogens11010095 (PMC8778306; doi:10.3390/pathogens11010095)

Supplementary Figure S1. Competitive ELISA assay. The figure shows the results of the reaction against the specific peptide HERV-K $env_{109\text{-}126}$ of a plasma of a positive patient sample that has been pre-incubated with: no peptide, a different positive peptide (Annexin A2 $_{13\text{-}37}$ LEGDHSTPPSAYGSVKAYTNFDAER) and the peptide of interest (HERV-K $env_{109\text{-}126}$). *(The experiment was performed in triplicate)

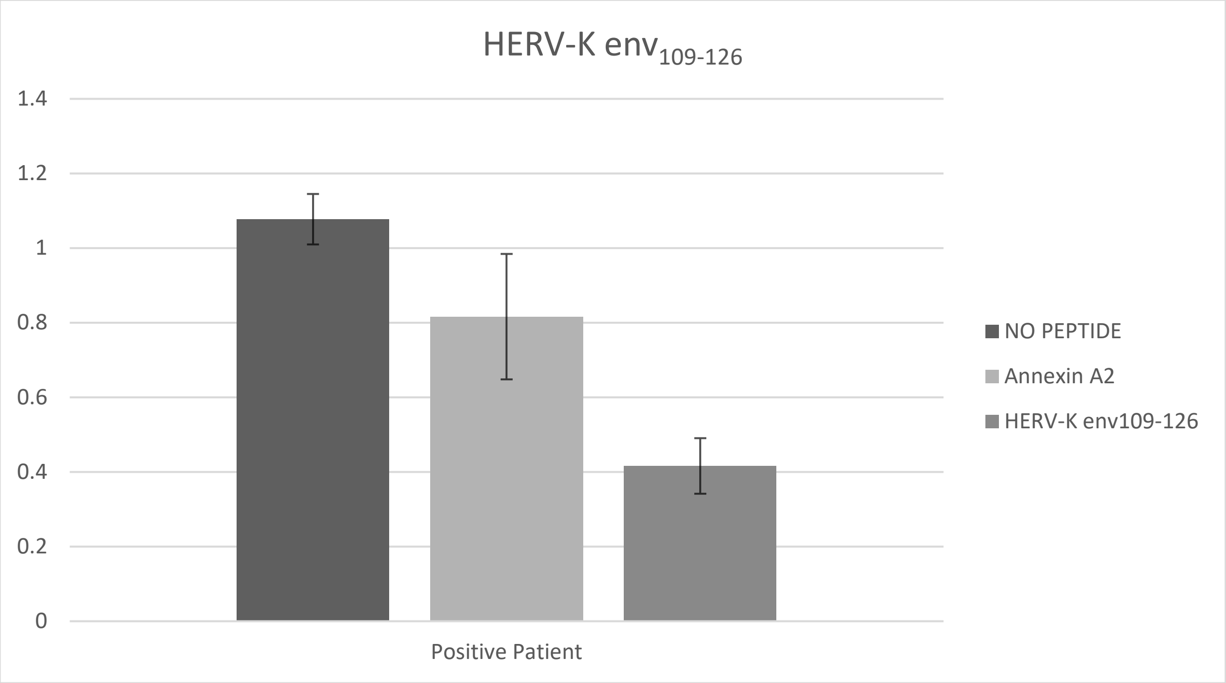

Supplement: Supplementary file 1 [file pathogens-11-00095-s001.zip › pathogens-1521461-supplementary.pdf]
